# Precursor-induced conditional random fields: connecting separate entities by induction for improved clinical named entity recognition

**DOI:** 10.1186/s12911-019-0865-1

**Published:** 2019-07-15

**Authors:** Wangjin Lee, Jinwook Choi

**Affiliations:** 1 Interdisciplinary Program for Bioengineering, Graduate School, Seoul National University, 103 Daehak-ro, Jongno-gu, Seoul, 03080 South Korea; 2 Department of Biomedical Engineering, Seoul National University College of Medicine, 103 Daehak-ro, Jongno-gu, Seoul, 03080 South Korea; 3 Institute of Medical and Biological Engineering, Medical Research Center, Seoul National University, 101 Daehak-ro, Jongno-gu, Seoul, 03080 South Korea

**Keywords:** Clinical named entity recognition, Conditional random fields, High-order dependency, Clinical natural language processing, Induction method

## Abstract

**Background:**

This paper presents a conditional random fields (CRF) method that enables the capture of specific high-order label transition factors to improve clinical named entity recognition performance. Consecutive clinical entities in a sentence are usually separated from each other, and the textual descriptions in clinical narrative documents frequently indicate causal or posterior relationships that can be used to facilitate clinical named entity recognition. However, the CRF that is generally used for named entity recognition is a first-order model that constrains label transition dependency of adjoining labels under the Markov assumption.

**Methods:**

Based on the first-order structure, our proposed model utilizes non-entity tokens between separated entities as an information transmission medium by applying a label induction method. The model is referred to as precursor-induced CRF because its non-entity state memorizes precursor entity information, and the model’s structure allows the precursor entity information to propagate forward through the label sequence.

**Results:**

We compared the proposed model with both first- and second-order CRFs in terms of their F_1_-scores, using two clinical named entity recognition corpora (the i2b2 2012 challenge and the Seoul National University Hospital electronic health record). The proposed model demonstrated better entity recognition performance than both the first- and second-order CRFs and was also more efficient than the higher-order model.

**Conclusion:**

The proposed precursor-induced CRF which uses non-entity labels as label transition information improves entity recognition F_1_ score by exploiting long-distance transition factors without exponentially increasing the computational time. In contrast, a conventional second-order CRF model that uses longer distance transition factors showed even worse results than the first-order model and required the longest computation time. Thus, the proposed model could offer a considerable performance improvement over current clinical named entity recognition methods based on the CRF models.

## Background

With the recent application of artificial intelligence to the medical field, health information systems are expected to handle medical data in the form of unstructured text. The unstructured clinical text conveys descriptions of patients’ health information, including their histories of illness and hospital treatment. Salient concepts that express a patient’s health status are represented by named entities (NEs) in the text. The identifying textual mentions of health-related concepts, termed clinical named entity recognition (NER), is a sub-problem in the field of clinical natural language processing (NLP) [1]. The health information that requires identification can range from a single entity to an elaborate description containing many entities. Heterogeneous classes of clinical entities have been employed in recent studies; these are strongly related to clinical activities, such as medical examination, medication, and diagnosis [2–7].

**Fig. 1:**
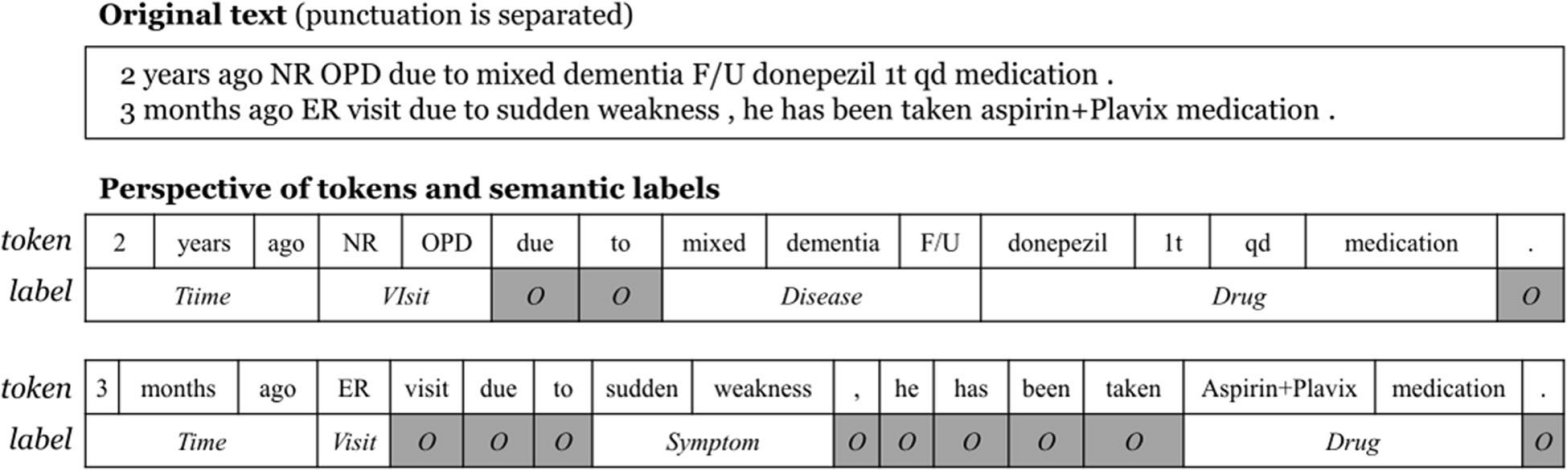
NER perspective of a text; the label *O* represents a non-entity

The NER problem consists of identifying spans of entities and attaching labels indicating the appropriate semantic class, as shown in Fig. 1. In the NER task, the text can be seen as a word (or token) sequence, and the most advanced NER models are therefore based on sequence labeling approaches that use machine learning methods [3, 4, 8, 9]. The concept of conditional random fields (CRFs) [10] has demonstrated promising results in many sequence labeling problems, including NER [3, 10–14], as well as a deep learning architecture applied to the NER task [15, 16]. CRF models are particularly effective for text processing because they learn transition factors between labels of single tokens, assuming that the current label is conditioned on both current observations and the immediately preceding label. The first-order constraint is applied in order to reduce computational complexity and to maintain the model’s simplicity.

However, the constraint on the labels’ adjacency prevents the model from expressing transition dependency between entities separated by a long distance. NEs tend to be separated by non-entity words in NER problems, and this innate attribute inhibits the first-order CRF model’s ability to capture dependencies between NE labels when the two entities are separated by non-entities that are outside tokens [17]. Therefore, CRF models using the first-order transition factor have difficulty in capturing higher-order interdependencies of NEs.

More specifically, we assumed that 1) named entities are prevalent in clinical texts, 2) the entities in clinical texts are semantically related, thus the information of preceding entity’s label would be an important feature for an NER model’s prediction of a certain label (at a time step), 3) the labeling mechanism of the CRF model that uses label transition information as one important feature would be suitable for clinical NER. However, according to the study published by Dan Roth’s group (CoNLL 2009), it is limited to use the transition information, especially for NER in the first- or second-order model based on Markov assumption because the named entities are generally separated each other in a text.

Previous NER studies have focused on methods of exploring long-distance dependencies in NER while maintaining computational tractability. Conventional high-order CRFs is known to be intractable in practice because they multiply the feature space and require more training data to prevent the data sparseness problem [18]. Sarawagi and Cohen proposed a semi-Markov CRF [19] that treated the same consecutive labels as a segment and used the label transition between adjoining segments. Subsequent studies have proposed using pre-defined label patterns to implement high-order CRFs [20–22]. However, these methods suffer from limitations associated with the management of entity transitions within non-entity labels of arbitrary length.

This study focuses on using the interdependency of NEs separated by an arbitrary number of non-entity tokens, a condition that is predominant in clinical texts but rarely captured by first-order CRF models. In order to minimize the increase in the model’s computational complexity associated with the extraction of long-distance label transition information, this study proposes an induction method that allows information to propagate from one state to state between two entities through non-entity sequence within a single instance.

Concentrating on the CRF model study rather than the medical NER, this paper would briefly introduce recent studies in medical NER. Deep-learning based methods for clinical concept identification are actively studied especially based on recurrent neural network structures [16, 23–28]. In the long short-term memory and CRF architecture, the CRF is still used for labeling of a sequence because the CRF model can jointly use neighboring tags in its output decision [15]. In order to automate medical NER a research [29] has been proposed to incorporate active learning. Once named entities are extracted, the identified terms can be utilized in order to derive more information beyond textual data, such as temporal information extraction [3, 30], drug-disease relationship recognition from large scale medical literature [31], and identification of risk factors related to a particular disease [32]. In order to support researchers requiring NER modules, off-the-shelf medical NER programs are recently published such as CLAMP [33] and MetaMap Lite [34].

The remainder of this paper is organized as follows. The Methods section details the proposed CRF model and the model evaluation method. The Results section presents the evaluation results, and the Discussion section considers several observations related to the use of the proposed model in clinical NER. The Conclusion section summarizes the study’s main findings.

## Methods

### Conditional random fields

In the conventional CRF model applied to NER, a textual instance (i.e., sentence) can be represented as a pair (***x***, ***y***) where ***x*** is an observed feature sequence including one or more words (tokens) and ***y*** is the feature sequence’s corresponding label sequence. Because the text is a linear sequence of tokens, the CRF for NER takes the form of a linear chain. The length of ***x*** is the number of tokens, and the sequence ***y*** has the same length as ***x***. The label is hidden, and a hidden state value set consists of the target entity labels and a single non-entity label for non-entity tokens. The CRF model then represents the conditional distribution *P*(***y***|***x***) as an equation of feature functions as follows:

1 $$ \mathrm{p}\left(y|x\right)=\frac{1}{Z(x)}\bullet {\prod}_{t=1}^T\mathit{\exp}\left\{{\sum}_{k=1}^K{\theta}_k{f}_k\left({y}_t,{y}_{t-1},{x}_t\right)\right\}\kern0.75em ,\kern0.5em $$

where *f*_*k*_ is a *k*^th^ arbitrary feature function having the corresponding weight *θ*_*k*_, *K* is the number of feature functions, *t* is the time step, *T* is the number of tokens in an instance of *x*, and *Z*(*x*) is a partition function summing the numerator for all possible *y* sequences [35]. The learning objective is to find the weight set that maximizes the conditional distribution. The function *f*_*k*_ is a binary indicator function that has a value of 1 only if the function matches the target condition, and is otherwise 0. Dependencies between random variables are presented in the form of feature function *f*_k_ in the CRF; the feature functions are either transition factors or observation factor functions. The transition factors in the CRF model take the form of *f*_*k*_^ij^(*y*, *y’*, *x*) = **1**_{y = i}_**1**_{y’ = j}_ where *i* and *j* are certain label symbols having transition relationship according to this function. The observation factors takes the form as Eq. (2) where *i* and *o* are certain symbols having an explicit relationship according to this function:

2 $$ {f_k}^{\mathrm{io}}\left(y,y',x\right)={\mathbf{1}}_{\left\{\mathrm{y}=\mathrm{i}\right\}}{\mathbf{1}}_{\left\{\mathrm{x}=\mathrm{o}\right\}}.. $$

**Fig. 2:**
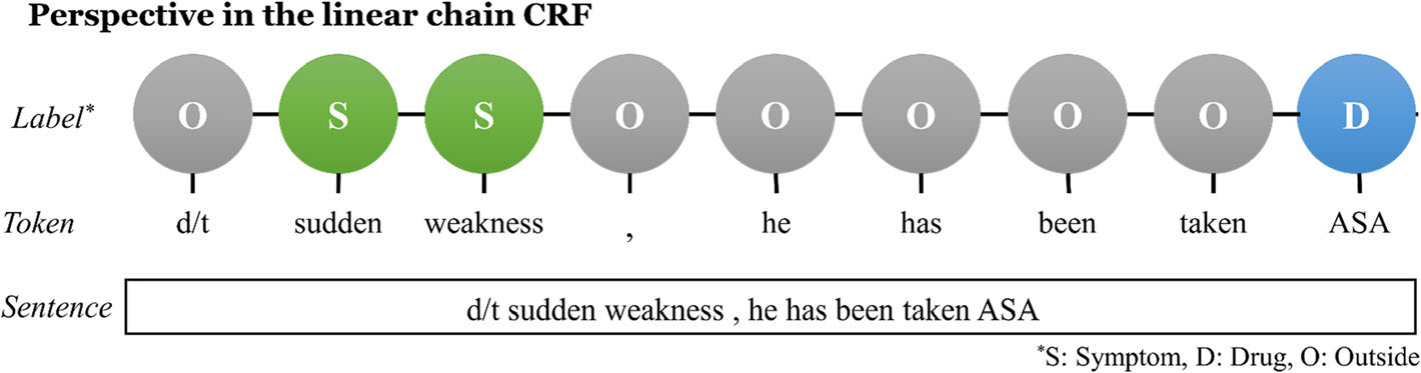
Example of entities separated by non-entity words in the CRF model (*S*: symptom; *D:* drug; *O:* non-entity)

Based on this definition of the feature function, the CRF model explicitly represents not only observation information but also label transition information for sequence labeling. For instance, presume a set {*A*, *B*, *O*} as the label symbol set; assign *A* or *B* to NEs, assign a label symbol *O* to non-entity tokens, and presume a label sequence of length 4, [*A*, *B*, *O*, *B*], where the first occurrence of entity *B* follows entity *A*, and a single non-entity token exists between the two entity *B*s. The first-order CRF models only those label transitions between adjoining state labels, that is, the label transition data {(*A*, *B*), (*B*, *O*), (*O*, *B*)}, in which the transition between labels *A* and *B* is explicitly expressed. Presume another label sequence [*A*, *O*, …*O*, *B*] where entity *A* precedes entity *B* by some distance and an arbitrary length of consecutive non-entity tokens are between the two NEs. The first-order CRF model learns only the label transitions {(*A*, *O*), (*O*, *O*), (*O*, *B*)} from the data, in which the dependency (*A*, *B*) is not explicitly captured by the model and the fact that entity *A* precedes entity *B* is not learned during the training time. Because the CRF model treats single observation tokens as single time steps in a sequence, the gap size between two separate entities is broadened by the number of intermediary non-entities, as shown in Fig. 2.

In Fig. 2, each circle denotes a random variable for labels, and each edge denotes that there is a dependency between connected random variables. In this structure, labels have dependency only between neighbors. Thus a dependency for entity prediction between the label symbols ‘Symptom’ and ‘Drug’ for predicting the word ‘ASA’ seems to be ignored. In the case of the ‘ASA,’ we suspected that the preceding label information could provide additional information for prediction of a particular label for the word if the information can be delivered forward.

### Precursor-induced conditional random fields

**Fig. 3:**
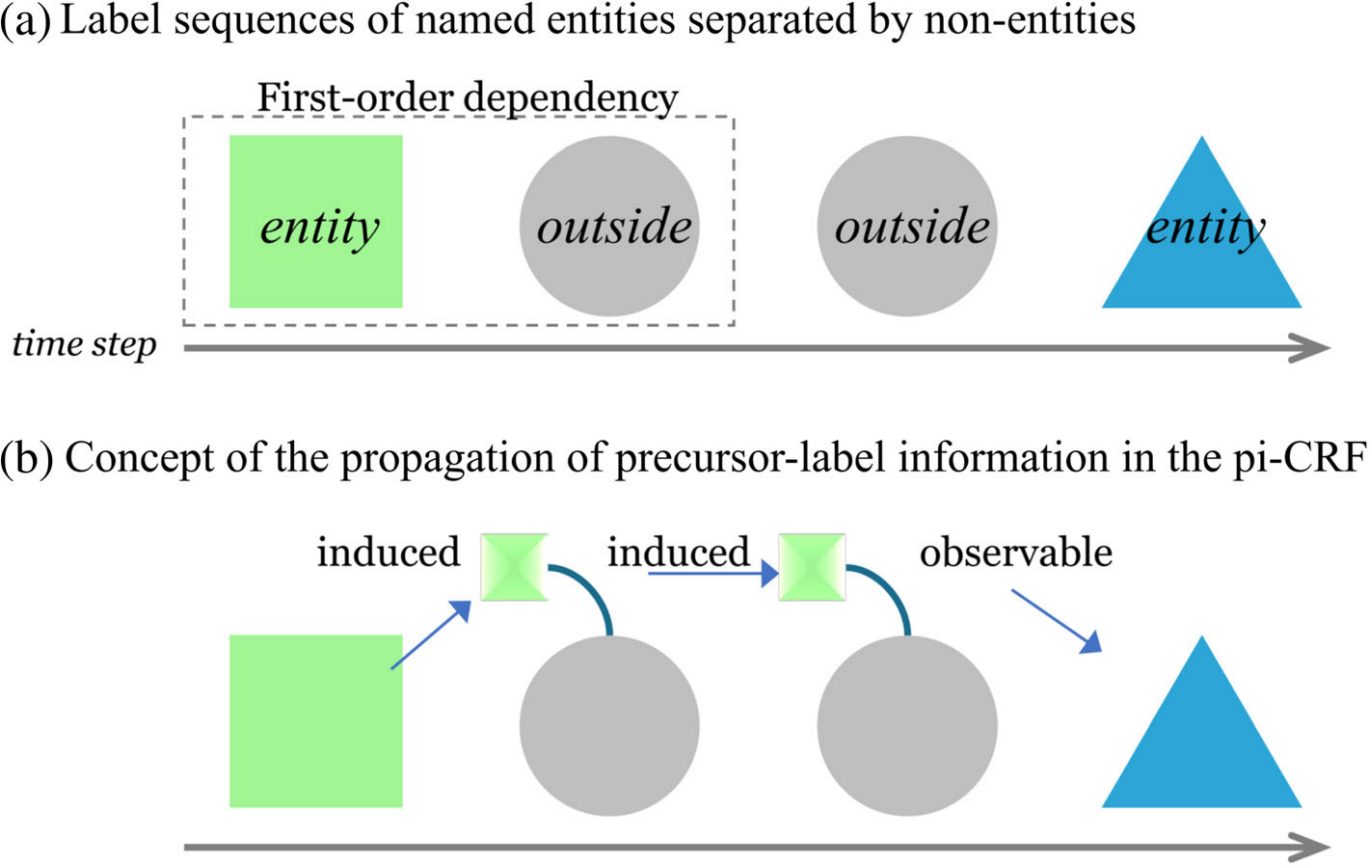
The transformation from conventional first-order CRF to precursor-induced CRF

In order to improve the CRF model for NER applications, this study introduces a precursor-induced CRF (pi-CRF) model to capture specific long-distance transition dependencies between two NEs separated by multiple non-entities. The pi-CRF model:

- Uses non-entity labels to propagate transition information between separated NEs;
- Retains the first-order model structure to reduce the model’s computational complexity than the second-order or higher-order CRF;
- Focuses on label subsequences with the [*entity*, *outside*^+^, *entity*] pattern, as shown in Fig. 3 (a), where the *outside*^+^ notation denotes one or successive non-entity label symbols;
- Adds a memory element to the hidden state variables to represent those states labeled as non-entities, such that the initial *outside* label in a non-entity subsequence propagates its explicit first-order dependency on its adjacent *entity* to the next *outside* label, which in turn propagates the information to the next *outside* label, as shown in Fig. 3 (b); and
- Uses an induction process to transmit the information from the first *entity* through multiple *outside* label sequence to the second *entity* state, even though the model uses the first-order dependency (Fig. 3 (b)).
- Modifies the observation feature functions of the CRF in order to share observation symbols among *outside* label symbols (Eq. 4).

### Label induction

In the pi-CRF, a state with an *outside* label binds with an additional memory element and behaves as an information transmission medium, delivering information about the presence or absence of the preceding entity forward, which requires the expansion of the hidden state value set (label symbols). The entity label symbols are collected from the training data, and the expanded state value set is eventually derived by a concatenation of entity label symbol and the *outside* label symbol. The concatenated *outside* label symbols thus indicate that the *outside* label follows a specific entity label. As a naming convention, we use *label*[O]^+^ to implicitly indicate that the sequence of *O* (*outside*) labels follows the concatenated label series. In the example, the symbol *A*[O]^+^ is one *outside* label symbol that indicates that an entity *A* precedes itself, and *O*[O]^+^ is one fragmented *outside* label symbol indicating that no entity has occurred before this non-entity state. The CRF models distinguish the features for observation symbols and the label symbols. Thus, any types of label symbols do not violate the token symbols, and any label naming convention can be used.

The form of the pi-CRF is derived from Eq. (1), and the conditional probability distribution of the CRF model extension takes the form of feature functions as follows:

3 $$ \mathrm{p}\left(y,a|x\right)=\frac{1}{Z(x)}\bullet {\prod}_{t=1}^T\mathit{\exp}\left\{{\sum}_{k=1}^K{\theta}_k{f}_k\left({y}_t,{y}_{t-1},{x}_t,{a}_t,{a}_{t-1}\right)\right\}, $$

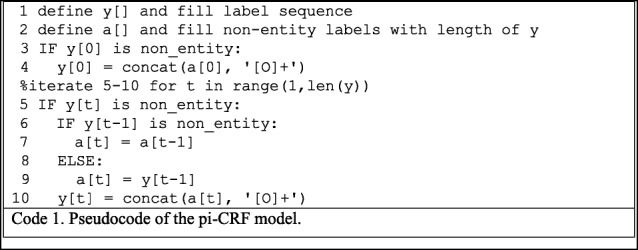

where the variable *a* stores the induced label information, and the value of *a*_t_ is activated by the value of *a*_t-1_ and y_t_. The conjoined variables *a* and *y* are eventually used to derive a newly induced label sequence: once *a*_t_ is activated, *a*_t_ transmutes the value of y_t_ (see the Code 1). Based on this model, the dependency of label transition is engaged within only adjacent tokens (i.e., y_t_ and y_t-1_) because this model is designated to keep the first-order structure. Thus, the information exists flows forward with the induced outside label by the first-order transition. This structure makes the conveyed information flows forward regardless of the distance.

**Fig. 4:**
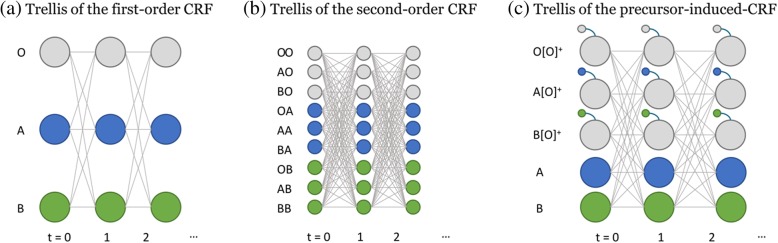
Trellis graphs generated by different CRFs; each circle indicates random hidden state variables at each time step, and lines indicate the transition paths among the labels. The small circles in (**c**) are the memory elements added to the hidden states for the non-entity label

This induction process subsequently expands the original label symbol set inside the model, producing newly induced and multiple *outside* label symbols instead of the single *outside* label symbol. For example, the process modifies an original label sequence [*A*, *O*, ⋯*O*, *B*] to [*A*, *A*[*O*]^+^, ⋯*A*[*O*]^+^, *B*] according to Code 1. This transformation helps the model learn long-distance transitions between successive NEs even in the first-order form: from the modified example sequence, the model can learn label transition data {(*A*[*O*]^+^, *B*)} where the entity *B* depends on the *non-entity* taking *entity A* as its precursor. This process also generates a trellis structure (Fig. 4 (c)) that is slightly more complex than the trellis generated by the conventional first-order CRF model (Fig. 4 (a)), but simpler than the trellis generated by a conventional second-order CRF model (Fig. 4 (b)). The CRF models generally have as many hidden state options (represented by the nodes in Fig. 4) as there are variables at each time step, and each combination of hidden states denotes a path forward. If *N* is the number of hidden states in the original first-order CRF model, the pi-CRF model introduces *N* additional new states; however, this increase in computational complexity is relatively moderate compared to the increase induced by second- or higher-order CRF models. In addition, if the IOB2 tagging scheme [36] is applied to the pi-CRF model, the increase in the number of newly induced hidden states is halved.

One of the main factors determining the CRF model’s complexity is the model’s graphical structure. The structure can be presented in the form of a tuple. Thus, the structures of the first-order CRF can be presented in (*y*_t-1_, *y*_t_, *x*_t_). Because the relationship between *y*s is related to transition, the number of transition pair (*y*_t-1_, *y*_t_) can be *N*^2^. It means that at least *N*^2^ calculations are required for each time step of a sequence in both of the training and testing time. In the same way, the graphical structure of the second-order CRF can be presented in (*y*_t-2_, *y*_t-1_, *y*_t_, *x*_t_) and the transition pair (*y*_t-2_, *y*_t-1_, *y*_t_) derives at least *N*^4^ (=*N*^2^ times *N*^2^) calculations for each time step in training and testing the second-order model. According to the formulation of the pi-CRF (Eq. 2), the variable *a* does not act as a hidden variable but interacts with the variable *y* in order to expand the possible values of the variable *y.* This system allows the pi-CRF to operate in the first-order structure and it keeps the model’s complexity feasible.

### Observation symbol sharing

It is worth addressing one of the attributes of the pi-CRF. The model uses modified observation feature functions. The observation feature function *f*_k_^io^ (Eq. 2) directly implies that a certain label *i* has ‘one-to-one’ relationship with a certain observation symbol *o*. If a label symbol does not have a relationship with a particular observation symbol, its relationship is not trained.

The label induction process makes multiple outside label symbols (i.e., ‘*label*[O]+’ symbols), instead of using one single outside symbol (i.e., ‘O’ symbol for the outside label). This induction process would interrupt an outside label symbol to have relationships with whole observation symbols related to non-entities.

Finally, each outside label symbol has relationships with only a portion of observation symbols. For the same training data, it is generally known that machine learning models with more hidden states are more likely to experience data sparseness problems because of their increased feature dimensions [37]. Likewise, in our development period, we observed that the first-order CRF performs worse if the conventional model was trained with the induced label pattern.

In order to prevent the performance decrease, the multiple *outside* symbols are allowed to share an observation symbol each other in the pi-CRF model, according to the following observation feature function:

4 $$ {f_k}^{io}\left(\mathrm{y},{\mathrm{y}}^{\prime },\mathrm{x}\right)={\mathbf{1}}_{\left\{x=o\right\}}\bullet \left({\mathbf{1}}_{\left\{i\in \neg outside\ and\ y=i\right\}}+{\mathbf{1}}_{\left\{i\in outside\ and\ y\in outside\right\}}\right) $$

The second and the third indicator terms in the right-hand side determine whether the *y* value is an outside label symbol or not. If the *i* (the corresponding label symbol of the function *f*_k_) is not outside symbol, then this equation tests whether the *y* value is equal to *i*. Contrary, if the *i* is an outside symbol, then the third indicator term has value 1 as long as the value of the *y* is an outside symbol. Unlike the feature functions in the conventional CRF constrain ‘one-to-one’ relationship between a label symbol and an observation symbol in a feature function, the third indicator term allows ‘many-to-one’ relationship between whole outside label symbols and one observation symbol.

In the pi-CRF, the model used the Eq. (4) for its observation feature function instead of using the Eq. (2) that is used in the conventional CRF. By way of illustration, presume a token, “doctor,” occurred with three outside label symbols (O[O]^+^, A[O]^+^, and B[O]^+^) in the training set. According to the definition of the observational feature function constraining one-to-one relationship, a first-order CRF has three distinct feature functions *f*_a_^io^(*x* = doctor, *y* = O[O]^+^), *f*_b_^io^(*x* = doctor, *y* = A[O]^+^), and *f*_c_^io^(*x* = doctor, *y* = B[O]^+^). Although the original CRF treats the three feature functions independently, the pi-CRF has one single feature function for the observation symbol and the outside label symbols, for instance, *f*_k_^io^(*x* = doctor, *y* = *outside symbol*).

### Model implementation

Both the original and the pi-CRF models were implemented using Java. The basic CRF structure and algorithms were implemented in MALLET [38]. The pi-CRF model was trained using the original linear chain CRF algorithms without modification because the graphical architecture of the pi-CRF model is fixed as a template for each time step in the same manner as in the original CRF model. In order to train the pi-CRF model, the L-BFGS optimization method [12] and *l2*-regularization [39] were used to exploit the conventional CRF model’s most advantageous features [35]. Furthermore, the Viterbi algorithm was used for inferences from unlabeled sequences. The executable files are available online.^1^

### Parameter tuning

In order to train both models properly, the model parameters were regularized during the development phase. In both the original and the pi-CRF models, *l*2-regularization [39] was used in order to avoid overfitting, and the form of regularization is as that in Eq. 5:

5 $$ -{\sum}_{k=1}^K\frac{\theta_k^2}{2{\sigma}^2},\kern0.5em $$

where *K* is the number of feature functions and *θ*_*k*_ is the weight of the *k*^th^ feature function *f*_k_, and σ is the hyper-parameter for the regularization that adjusts the amount of penalty. The regularization term is applied to a log-likelihood form of the CRF models and penalizes large weights.

During the model development process, the training data were split by 8:2 for each training and development set and the parameter σ was chosen to provide the best F_1_-score for the development set. The parameter tuning was independently performed on each data set, and the third feature set was used during the tuning process.

## Results

### Dataset description

All the experiments were performed on the NER sets in clinical and general domains: English clinical texts (i2b2 2012 NLP shared task data [3]), rheumatism patients’ discharge summaries obtained from Seoul National University Hospital (SNUH) [40], and the CoNLL-2003 NER shared task corpus [41]. The documents in the SNUH set were written using English and Korean. The discharge summaries were annotated using the IOB2 tagging scheme [36].

Although the original annotation in the i2b2 2012 data contains more semantic classes, this evaluation was conducted using the *problem*, *test*, and *treatment* entities. For the SNUH corpus, the entities of *symptom*, *disease*, *clinical lab test*, *medication*, and *procedure/operation* were used. We are interested in identifying clinical events related to a patient’s clinical events. Thus, we used the clinical semantic classes listed above in our evaluation. For the CoNLL-2003 data, the entities of *location*, *person*, *organization*, and *miscellaneous* were annotated from the general domain news articles.

Tables 1 and 2 show the data and annotation statistics for each data set. The training and testing sets in the i2b2 2012 and the CoNLL-2003 NER sets were divided following the official distribution set by the data source administrators.

**Table 1:** Data specification

Corpus	Domain	Set	Article	Sentence	Token	Entity
i2b2 2012	Clinical	Train	190	7,258	94,836	11,239
		Test	120	5,547	78,564	9,623
SNUH	Clinical	Train	196	11,669	116,402	18,383
		Test	193	11,042	107,666	17,125
CoNLL 2003	General	Train	946	14,987	203,621	23,499
		Test	231	3,684	46,435	5,629

**Table 2:** Annotation statistics

a) i2b2 2012					
Set	Problem	Test		Treatment	
Train	4,962	2,558		3,719	
Test	4,270	2,140		3,213	
b) SNUH					
Set	Symptom	Test	Disease	Medication	Procedure
Train	3,923	4,559	5,084	3,642	1,175
Test	3,737	3,917	4,828	3,496	1,147
c) CoNLL 2003					
Set	Location	Person	Organization	Miscellaneous	
Train	7,140	6,600	6,321	3,438	
Test	1,656	1,617	1,662	694	

**Fig. 5:**
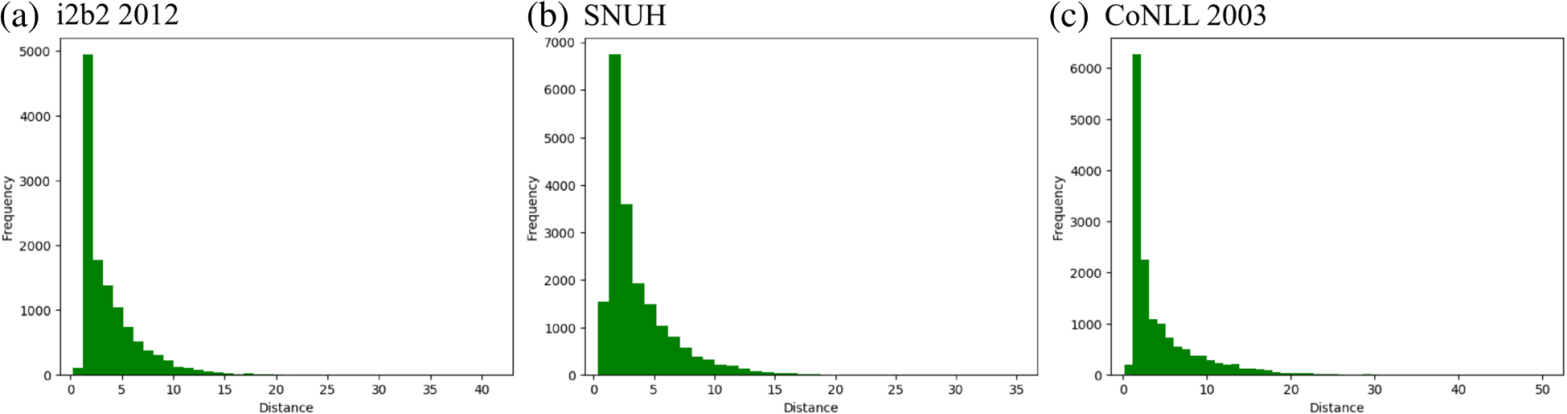
Histograms of distances between named entities in each corpus. The number ‘n’ on the x-axis means n non-entities exist within the two entities

As we assumed that a significant portion of the NEs is separated in sentences, we measured the word distance between the entities in the data sets. The distance dependency was measured within each instance. Table 3 shows examples of the distances between entities in the i2b2 corpus and Fig. 5 shows the distributions of distances between entities in the entire data set for each corpus. The median distance value between entities was 3 and the mean values were within the range of from 3 to 5, indicating that the NEs in the data sets tended to be separated by 3 to 5 non-entity tokens. The data also indicates that the number of entities within the first-order range is less than the number of entities within the second- or higher-order ranges. In addition, the ratios of the number of entities having transition dependency to the total number of entities were 0.85, 0.73, and 0.78 for i2b2 2012, SNUH, and CoNLL2003 data sets, respectively. These values indicate that in most cases, entities tend to be interrelated in an instance, rather than being present as single entities.

**Table 3:** Example sentences of the entity distances (single: entity not having a precursor)

Type	Example sentence with entity annotation
single	The patient is a 28-year-old woman who is [HIV positive]_problem_ for 2 years .
distance 0	With [intravenous hydration]_treatment_ [the BUN]_test_ and …
distance 1	… because of [pancytopenia]_problem_ and [vomiting]_problem_ on [DDI]_treatment_
distance *8*	She was brought in for [an esophagogastroduodenoscopy]_test_ on 9/26 but she basically was not sufficiently [sedated]_treatment_ and readmitted at this time for [a GI work-up]_test_ .

### Feature settings

Three types of feature settings were investigated in this evaluation, as summarized in Table 4. The setting #1 is the simplest available, and the setting #2 is the configuration in which character-wise prefixes and suffixes could be exploited. Although these two settings use only simple features, these configurations reduce the potential bias that the features could exert on the performance comparison. The setting #3 implemented features used in previous evaluations of NER methods for each data set [17, 40, 42]; some particular features that are easy to implement were selected for use here. Also, “Token” and “n-gram” are typical features used in NER. The morphologic information used included character-wise affixes (i.e., the first two characters of a token), capitalization patterns (e.g., all capitalized or capitalization at the word beginning) [17]. Matching indicates whether a token matches a controlled vocabulary, e.g., the previous token is an obvious modifier of the current token, or a token is matched to a list consisting of the first entity tokens in the training data (frequency > 10) as performed by Li, et al. [43].

**Table 4:** Summary of the feature settings. (The w denotes the window size. If the value is absent, only feature of the current token is used. The n denotes the n of the n-gram. The ‘len’ denotes the length of affixes. The matching features denote the result of controlled vocabulary matching)

Set	Token	Norm-token	n-gram	character affix	capitalization	POS/Chunk	Matching
#1-context	w = 3	w = 3					
#2-morph	w = 3	w = 3		le*n* = 2~3 w = 3			
#3-i2b2	w = 5	w = 5	n = 2 w = 5	len = 2~7 w = 3	w = 1		
#3-snuh	w = 5	w = 3	n = 2 w = 5	len = 2~3			modifier /control
#3-conll	w = 5			len = 3~4 w = 5	w = 5	*n* = 1	

### Performance evaluation

We used the three NER datasets to compare the proposed model structure with the first- and the second-order linear chain CRFs, and semi-Markov CRF [19], high-order CRF [18] that are variants of the CRF leveraging higher-order label transition dependency.

At first, we compared the pi-CRF with the first-order models. Table 5 shows the F_1_ scores of the first-order CRF, the first-order CRF trained with the induced labels, and the pi-CRF for each test set. F_1_ score is harmonic mean of the precision and recall scores. We first tested the models on all instances in each data set, and then tested the models on only those instances having two or more entities. The table shows that the proposed model structure offers a demonstrable improvement over the first-order models. The pi-CRF showed higher F_1_ scores for all feature settings on both the i2b2 2012 and the SNUH data sets.

**Table 5:** F_1_ scores of the first-order models and the pi-CRF for each corpora. The first value (‘whole instance’) is F_1_ score with whole test set and the second value (‘distanced instance’) is F_1_ score evaluated only with instances having transition dependency between NEs. (bold: best performance, shaded: pi-CRF)

Feature	Models	i2b2 2012		SNUH		CoNLL 2003	
		whole instance	distanced instance	whole instance	distanced instance	whole instance	distanced instance
Set 1	1st-order CRF	67.22	68.24	74.75	73.20	**60.68**	**62.19**
	1st-order CRF with induced labels	66.60	67.69	74.09	72.85	23.38	15.24
	pi-CRF	**67.29**	**68.43**	**75.50**	**74.43**	45.54	43.41
Set 2	1st-order CRF	71.61	72.85	75.81	75.04	68.43	**72.93**
	1st-order CRF with induced labels	70.73	71.98	75.24	74.36	44.90	41.89
	pi-CRF	**71.99**	**73.35**	**76.04**	**75.29**	**69.61**	72.31
Set 3	1st-order CRF	72.55	73.97	76.18	75.06	**82.57**	**83.13**
	1st-order CRF with induced labels	71.25	72.75	75.37	74.18	80.81	81.55
	pi-CRF	**72.58**	**74.04**	**76.24**	**75.33**	82.08	82.76

In addition, the first-order CRF with induced labels shows the worst performance than others. Even though the induced label patterns can be easily obtained in the first-order model, we can see that the use of the label induction without the ‘observation symbol sharing’ in the conventional model rather negatively affects its performance.

We also evaluated higher-order CRF models such as the conventional second-order CRF, semi-Markov CRF [19] and the high-order CRF [18, 20] implemented by A Allam and M Krauthammer [44]. The semi-Markov CRF and the high-order CRF are CRF variants using higher-order transition dependencies. The two CRF variants were trained with the stochastic gradient descent for 50 epochs. The results are reported in Table 6. As shown in the table, the pi-CRF shows a bit better performance than the other models in several settings and the pi-CRF also shows similar performance with the variants in a complex feature set.

**Table 6:** F_1_ scores of higher-order CRF models and pi-CRF for each corpora. The first value (‘whole instance’) is F_1_ score with whole test set and the second value (‘distanced instance’) is F_1_ score evaluated only with instanced having transition dependency between NEs. (bold: best performance, shaded: pi-CRF)

Feature	Models	i2b2 2012		SNUH		CoNLL 2003	
		whole instance	distanced instance	whole instance	distanced instance	whole instance	distanced instance
Set 1	2nd-order CRF	**69.46**	**70.88**	73.43	72.21	**58.34**	**54.52**
	semi-Markov CRF	67.87	68.91	73.44	71.61	37.31	34.13
	high-order CRF	68.38	69.52	73.50	71.69	36.97	33.87
	pi-CRF	67.29	68.43	**75.50**	**74.43**	45.54	43.41
Set 2	2nd-order CRF	70.99	72.31	74.31	73.27	**73.21**	72.26
	semi-Markov CRF	72.19	73.54	76.01	74.87	63.19	63.32
	high-order CRF	71.50	72.74	76.11	74.97	63.56	63.76
	pi-CRF	**72.30**	**73.61**	**76.20**	**75.47**	69.61	**72.31**
Set 3	2nd-order CRF	71.75	73.01	75.17	74.05	**83.13**	**83.96**
	semi-Markov CRF	69.30	70.73	76.70	75.79	82.47	83.29
	high-order CRF	69.26	70.64	**76.73**	**75.91**	82.18	82.80
	pi-CRF	**72.58**	**74.04**	76.28	75.45	82.08	82.76

In addition, we may observe the performance of the higher-order models including the pi-CRF were decreased in the general domain set (CoNLL 2003) in the simple feature settings. When we compare this result with the corresponding tests in Table 5, the pi-CRF performs worse than the conventional models for the CoNLL data, though, we may interpret the performance decrease of the higher-order models in naïve feature setting might be expected.

Table 7 compares the proposed model’s training and inference times using the feature setting #3 with the conventional models. The table shows the numbers of parameters, states, elapsed training time, training time per iteration, and elapsed inference time. These values indicate that the pi-CRF design was slightly more complicated than the first-order CRF, although the proposed design was less complicated than the second-order CRF while still exploiting the transition information between NEs separated by long and arbitrary distances.

**Table 7:** Efficiency test results. The numbers of parameters and states indicate the model’s size. The elapsed training/inference times indicate the model’s speed. (shaded: pi-CRF)

Data	Model	Parameter	State	Elapsed training time (sec)	Training time per iteration (sec)	Elapsed inference time (sec)
i2b2	1st-order CRF	442,705	8	1,550	12.5	1.7
	2nd-order CRF	581,604	64	6,819	55.4	5.7
	pi-CRF	442,768	11	3,751	17.0	2.1
SNUH	1st-order CRF	396,245	12	2,946	19.5	1.9
	2nd-order CRF	495,772	144	27,388	139.7	9.3
	pi-CRF	396,400	17	6,231	23.6	2.1
CoNLL	1st-order CRF	313,672	10	4,031	19.1	0.6
	2nd-order CRF	431,044	100	24,828	173.6	2.6
	pi-CRF	313,776	14	13,512	29.4	0.7

### Result analysis

We also examined the model’s behavior on the test data set. Table 8 shows the numbers of predicted entities and correct predictions on each held-out data set, using feature setting #1. For the clinical data sets, the models that used long-distance transition dependency (i.e., the second-order and pi-CRF) tended to predict more entities than the first-order model, and the pi-CRF model correctly predicted more entities than both the first- and second-order CRF models, resulting in an improvement in recall performance: + 0.7 and + 1.13 for the i2b2 and SNUH, respectively. The final F_1_-score of the pi-CRF was improved than the first-order model, and we may indicate that the improvement of the recall consequently affects the improvement of the F_1_-score of the pi-CRF. However, the models that used long-distance transition dependency (the second-order and the pi-CRF) showed the opposite behavior on the general data set, predicting noticeably fewer entities than the first-order model, although most of the higher-order models’ predictions were correct. Thus, the precision performance of the pi-CRF showed an improvement of + 16.4 for the CoNLL set, even though the recall performance was relatively low.

**Table 8:** The numbers of the models’ expectation and the correct on each held-out set. (shaded: pi-CRF)

Data	Model	Whole instances			Distanced instances		
		gold	expected	correct	gold	expected	correct
i2b2 (clinical)	1st-order CRF	9,623	7,361	5,708	8,552	6,188	4,927
	2nd-order CRF		7,785	6,046		6,547	5,245
	pi-CRF		7,542	5,775		6,397	5,012
SNUH (clinical)	1st-order CRF	17,125	15,326	12,128	12,520	10,813	8,540
	2nd-order CRF		15,702	12,053		11,088	8,524
	pi-CRF		15,516	12,322		11,012	8,758
CoNLL (general)	1st-order CRF	5,629	3,785	2,856	4,331	2,693	2,184
	2nd-order CRF		2,778	2,529		1,986	1,799
	pi-CRF		1,855	1,704		1,280	1,218

**Fig. 6:**
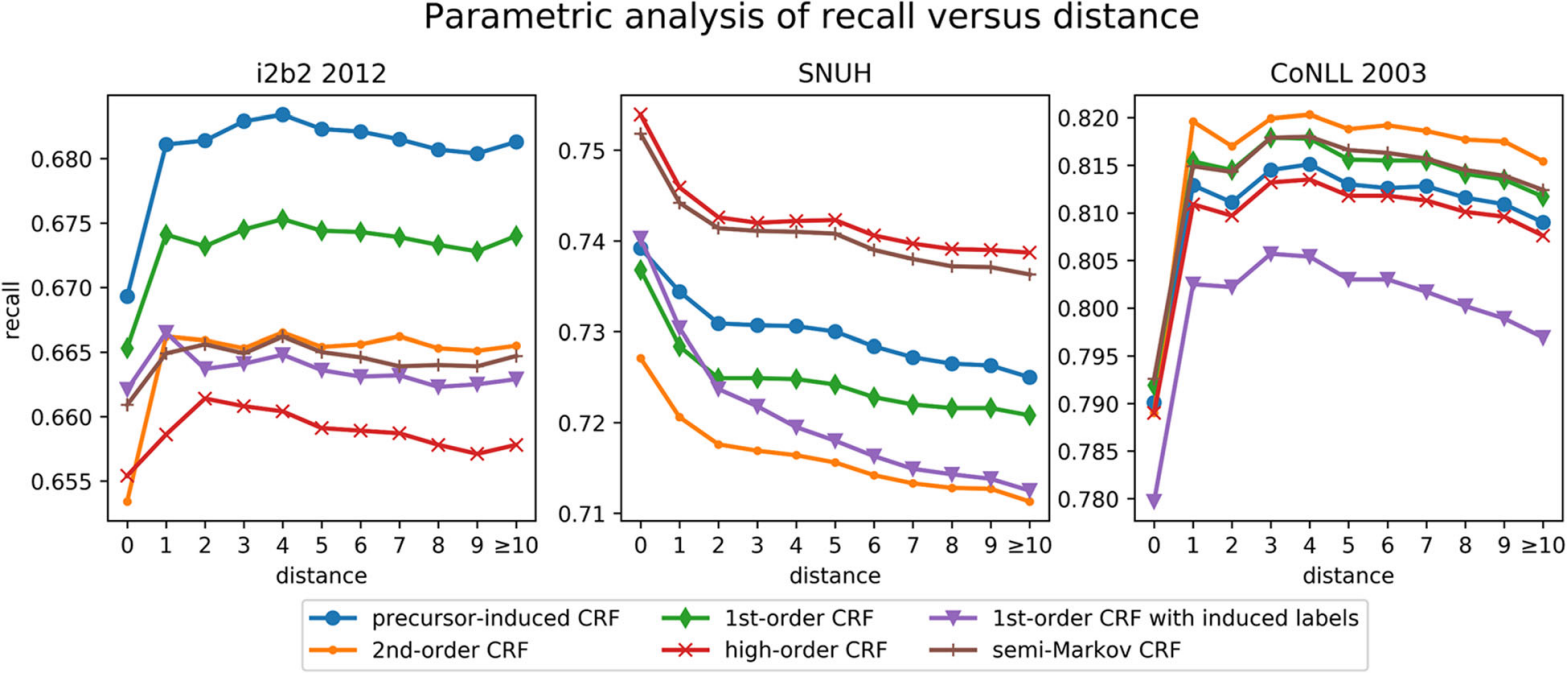
Recalls along the distances between named entities in each corpus. The y-axis denotes recall score, numeric labels on the x-axis denote sets of entities having outside labels between the entity and its precursors as much as the numbers. (feature set: set #3)

The models’ expectation performance were additionally analyzed along the distances from the preceding entities. Trying to analyze the models according to the distance between the entities, we inevitably used the recall. Because this evaluation of the models with recall alone has its limitations, so this result was presented as an auxiliary indicator. The initial recall scores were calculated only for the entities not having precursors, and then the recall scores were updated sequentially by adding entities along the distances from 0 to the maximum distance for each data set. Figure 6 shows the analysis result. The graph of the models moved similarly along with the distance between entities: according to this figure, we can observe the recall scores of the CRF decrease as distance increases. The CRF models seem to miss the entities following when two entities are consecutive. We could not observe a significant performance improvement of the pi-CRF compared to other models. However, the pi-CRF shows better results in this result when this model was compared with the first-order CRF that uses a similar graphical structure with the pi-CRF. Especially, the performance of the first-order model, which was trained with induced labels, was remarkably decreased according to the distance. The use of the induced label is easy in the conventional model, but, it would not guarantee the performance improvement in the model without the observation symbol sharing. The models’ recall scores have risen sharply at the points where distance is 1 in the i2b2 2012 and CoNLL. There is a small number of the entities having gap (order) value as 0 in both data collections: the numbers of entities having gap value as zero are 50, 30, and 707 in the i2b2, CoNLL, and SNUH data respectively.

## Discussion

In this study, we investigated the performance of the pi-CRF model which is a newly proposed variant of the CRF model designed particularly for extracting clinical NEs: the proposed model utilizes long-distance dependency relationships between the NEs separated by multiple non-entities in the CRF. The model fragments the non-entity state into fine-grained non-entity states and treats them as an information transmission medium based on the first-order linear chain CRF structure. The evaluation results showed that the proposed pi-CRF model is more effective at clinical NER. Although the pi-CRF model was slower than the first-order CRF, it was significantly faster than the second-order CRF model even while expressing higher-order transition dependencies between NEs.

Higher-order transitions are expressed as fixed-size label transitions in the conventional CRF model. Because the NEs tend to be separated by arbitrary distances, the conventional higher-order CRF model using a fixed-size state transition dependency has limited ability to express the desired information. One study of a semi-Markov CRF [19] proposed that consecutive units with the same label can be presented as a group although the model could not convey the information from the separated NEs. Based on this idea, we developed an induction method to present consecutive non-entity labels grouped by their precursor information. Besides, the mathematical formula (Eq. 3) used to express the proposed CRF model was derived from a CRF model that used virtual evidence [45], which incorporates prior knowledge of prototypes to make the model prefer to label consecutive values for a subsequence that matches a predefined pattern.

In contrast, our model used the formula to extend the hidden variables by joining two variables, *y* and *a*. The two hidden variables are conjoined in Eq. 3: the variables are multiplied, and they are merged into a new hidden variable instead of using two hidden variables in the mathematics form. Because the variable *a* has values only if the value of the corresponding *y* is the non-entity state, the multiplication implies that the newly derived hidden variable y’ has multiplied non-entity hidden states and the total number of the hidden states is expanded compared to the conventional CRF.

The design of the pi-CRF model improves the CRF model’s expressive power according to the evaluation results. The transition information is implemented as feature functions, and thus the transition information ultimately affects the model as one of many features. Leveraging the high-order label transition information, the pi-CRF shows better performance than other higher-order CRF models in many evaluation settings. It could be the model’s advantageous attribute that the proposed model preserves relatively compact model complexity than other higher-order models.

Avoiding the data sparseness problem was another significant concern in the model design. We expected the data sparseness problem to occur because the induction algorithm divides a single non-entity state into multiple states, and thus the frequency of observation features related to the outside label symbols was divided. In the model development phase, we observed that the model’s performance was inferior without the feature sharing implemented by Eq. (4). For the clinical NER tasks, the results showed that the pi-CRF design increased the F_1_ score compared with the first- and second-order CRF models while reducing the model’s speed loss. Further improvement could be achieved by testing models trained with more sophisticated features on various data sets, or porting the model onto the state-of-the-art neural NER architecture with long short-term memory [15].

## Conclusion

This study proposed a variant of the CRF model to improve the model’s expressive power for clinical NER problems, in which NEs tend to be separated from non-entities. The proposed pi-CRF utilizes non-entity labels between NEs as an information transmission medium that delivers the preceding entity information forward to the following entity. Our evaluation results showed that the proposed model improves clinical NER performance and reduces the computational complexity of the second-order CRF. Despite some inherent limitations, the results suggest that the utilization of non-entity labels could enable higher-order CRF model implementation while limiting the model’s complexity growth. We plan to test the model on various NER datasets and also to port the model onto a neural NER architecture [15] to further advance the clinical NER field.

## Data Availability

The executable Java file is available at the GitHub repository https://github.com/jinsamdol/precursor-induced_CRF. However, all data were extracted from the medical record of patients who had been admitted at SNUH, so the clinical data cannot be shared with other research groups without permission.

## Abbreviations

CoNLL: Conference on Natural Language Learning
CRF: Conditional Random Field
EHR: Electronic Health Record
i2b2: Information for Integrating Biology and the Bedsides
NE: Named Entity
NER: Named Entity Recognition
NLP: Natural Language Processing
pi-CRF: Precursor-Induced Conditional Random Fields
POS: Part-of-Speech
SNUH: Seoul National University Hospital

## Footnotes

1 The executable jar files are available in https://github.com/jinsamdol
